# Thrombospondin-1 Type 1 Repeats in a Model of Inflammatory Bowel Disease: Transcript Profile and Therapeutic Effects

**DOI:** 10.1371/journal.pone.0034590

**Published:** 2012-04-03

**Authors:** Zenaida P. Lopez-Dee, Sridar V. Chittur, Bhumi Patel, Rebecca Stanton, Michelle Wakeley, Brittany Lippert, Anastasya Menaker, Bethany Eiche, Robert Terry, Linda S. Gutierrez

**Affiliations:** 1 Department of Biology, Wilkes University, Wilkes-Barre, Pennsylvania, United States of America; 2 Center for Functional Genomics, University at Albany, State University of New York, Rensselaer, New York, United States of America; The Chinese University of Hong Kong, Hong Kong

## Abstract

Thrombospondin-1 (TSP-1) is a matricellular protein with regulatory functions in inflammation and cancer. The type 1 repeats (TSR) domains of TSP-1 have been shown to interact with a wide range of proteins that result in the anti-angiogenic and anti-tumor properties of TSP-1. To ascertain possible functions and evaluate potential therapeutic effects of TSRs in inflammatory bowel disease, we conducted clinical, histological and microarray analyses on a mouse model of induced colitis. We used dextran sulfate sodium (DSS) to induce colitis in wild-type (WT) mice for 7 days. Simultaneously, mice were injected with either saline or one form of TSP-1 derived recombinant proteins, containing either (1) the three type 1 repeats of the TSP-1 (3TSR), (2) the second type 1 repeat (TSR2), or (3) TSR2 with the RFK sequence (TSR2+RFK). Total RNA isolated from the mice colons were processed and hybridized to mouse arrays. Array data were validated by real-time qPCR and immunohistochemistry. Histological and disease indices reveal that the mice treated with the TSRs show different patterns of leukocytic infiltration and that 3TSR treatment was the most effective in decreasing inflammation in DSS-induced colitis. Transcriptional profiling revealed differentially expressed (DE) genes, with the 3TSR-treated mice showing the least deviation from the WT-water controls. In conclusion, this study shows that 3TSR treatment is effective in attenuating the inflammatory response to DSS injury. In addition, the transcriptomics work unveils novel genetic data that suggest beneficial application of the TSR domains in inflammatory bowel disease.

## Introduction

Thrombospondins (TSPs) are glycoproteins that are secreted into the extracellular matrix. They have important functions in development, inflammation, angiogenesis and cancer. The five members of this family (TSP-1 through 5) are all multimodular extracellular proteins. TSP-1 comprises a 450 kDA protein composed of three 150 kDA disulfide-linked polypeptide chains [1], [2]. TSP-1 and TSP-2 organize into trimeric structures. Each subunit of the trimer consists of multiple domains: an N-terminal globular domain, a procollagen domain, three types of repeated sequence motifs (type 1, type 2, and type 3 repeats) and a C-terminal globular domain. TSP-1 and TSP-2 both have the thrombospondin type 1 repeat, also called thrombospondin structural repeats (TSRs). TSP-3, TSP-4 and TSP-5, on the other hand, lack the TSRs and procollagen domain; they also differ from TSP-1 and TSP-2 in their pentameric structure. The thrombospondins have been characterized in a variety of organisms, including Drosophila, other arthropods and vertebrates [3].

The TSRs are about 60 amino acids in length and are evolutionarily conserved (e.g., [2], [3]–[5]). TSRs have roles in cell attachment and have been implicated in binding a variety of transmembrane and extracellular proteins. They have been shown also to have functions in the regulation of cell proliferation, migration and apoptosis in a variety of physiological and pathological events, such as wound healing, inflammation and inhibition of angiogenesis [6]. For example, by interacting with CD36 [7], [8] and integrins [9], TSRs inhibit endothelial cell migration.

The various functions of the TSRs have been attributed to several recognition motifs. Characterization of these motifs has led to the use of recombinant proteins that contain these motifs; these recombinant proteins are deemed useful in cancer therapy [10], [11]. The TSP-1 3TSR (that is, all three TSRs of the type 1 repeat domain) can activate transforming growth factor beta 1 (TGFβ1) and inhibit endothelial cell migration, angiogenesis, and tumor growth [10], [12]. In an efficacy study of 3TSR on human pancreatic cancer cells, 3TSR reduced tumor volume by 69% and induced extensive necrosis after 3 weeks of treatment. 3TSR treatment also reduced tumor microvessel density and increased apoptosis in the endothelia of tumors [12].

The TGFβ1 activating sequence RFK is located between the first and second TSR. When a squamous cell carcinoma cell line transfected with TSR2+RFK was injected into nude mice, TSR2+RFK inhibited *in vivo* tumor angiogenesis and growth in the mice. The tumors were shown to have increased levels of active TGFβ1. Treatment with TSR2 without RFK produced tumors that were slightly larger than the 3TSR and TSR2+RFK tumors [13]. However, mimetic peptides derived from TSR2, such as ABT-510 [14], decreased microvessel density and increased apoptosis in gliomas. ABT-510 was also effective in diminishing inflammation and angiogenesis in a murine model of inflammatory bowel disease (IBD) [15].

In this study, we evaluated the therapeutic effects of the subdomains of the TSP-1 TSRs using the DSS model of colitis and used a microarray approach to analyze the transcript profile of the mice treated with DSS and the TSRs. Our goals were to gain insight into the genes and pathways that are regulated upon TSR treatment and to determine the specific TSR treatment that would have the best potential for drug therapy. We were also interested in identifying novel genes not yet associated with and characterized in IBD, as well as putative intestinal stem cell genes. The results herein establish the efficacy of the 3TSR-containing recombinant protein in alleviating the inflammatory response to DSS injury as shown by improved clinical signs and lower perturbation of the transcriptome.

## Materials and Methods

### Mice

Wild-type (WT) mice (strain C57BL/6; n = 48) were purchased from Charles River Laboratories International (Wilmington, MA). Mice were bred at room temperature at the Wilkes University vivarium. All animal procedures were performed with the approval of the Wilkes University Institutional Animal Care and Use Committee and the U.S. National Institutes of Health (NIH) guidelines (Wilkes IACUC protocol # 189).

### Dextran sulfate sodium (DSS) and TSR Treatments

DSS (MW: 36,000∼50,000) (MP Biomedical, LLC, Aurora, OH) was dissolved in the drinking water (distilled) of the mice at a dilution of 2.5% (wt/vol) and administered to 6–7 week old mice for 7 days to induce acute colitis. Simultaneously, mice were subcutaneously injected daily with the following recombinant proteins: 3TSR (n = 10), TSR2 (n = 9) TSR2+RFK (n = 15). TSR doses were administered according to published results [10]. As a control for the TSR treatment, mice (n = 14) were injected with saline. Following 7 days of DSS administration, mice were sacrificed by CO_2_ asphyxiation.

Recombinant proteins were obtained from Dr. Jack Lawler (Harvard Medical School, Boston, MA). The 3TSR contains amino acids 361–530 of TSP-1; TSR2, amino acids 416–473; TSR2+RFK, amino acids 411–473 [10].

### Clinical parameters and Disease Activity Indices

The clinical severity of the colitis was determined daily by disease activity indices according to a published protocol [15]. Stool consistency was evaluated as follows: formed feces = 1, soft consistency = 2 and liquid feces = 3. Presence of fecal occult blood was tested using the stool guaiac test; scoring was done as follows: 1 for negative by guaiac paper, 2 for positive only with guaiac paper, 3 for bloody feces.

### Histology and inflammation grading in colonic tissues

Intestines were removed, opened longitudinally, and rinsed with ice-cold PBS. For morphologic studies, tissues were fixed in Histochoice MB fixative (Electron Microscopic Sciences, Hatfield, PA) overnight, processed and cut in serial 5-µm sections. Sections were stained with hematoxylin and eosin (H&E) for histopathological analysis. Inflammation was graded as follows: 0, no inflammation; 1, modest numbers of infiltrating leukocytes in the lamina propria; 2, infiltration of leukocytes leading to separation of crypts and mild mucosal hyperplasia; 3, massive infiltration of inflammatory cells accompanied by disrupted mucosal architecture and complete loss of goblet cells. Slides were double-coded before pictures were taken and frames blindly analyzed in a monitor.

### RNA isolation

Colon segments (∼1 cm long) were collected from treated and untreated mice and snap-frozen in liquid nitrogen. Total RNA was extracted from these tissues using the RNAqueous®-4PCR kit (Applied Biosystems), following the manufacturer's instructions. RNA was DNase I-treated and quantified using the Nanodrop ND1000 spectrometer (Thermo Scientific, Wilmington, DE). RNA integrity was checked on a non-denaturing agarose gel. The microarray experiments are described in the following sections following the MIAME guidelines.

### Microarray Processing

Total RNA from colons of at least three mice from each treatment group were submitted to the Center for Functional Genomics, University at Albany, Rensselaer, New York, for microarray processing. Treatment groups included the mice treated with DSS and the TSR-containing recombinant proteins 3TSR, TSR2 and TSR2+RFK and mice treated with DSS+ saline injections as controls. Untreated WT were used as additional control. RNA integrity of the samples used for microarray hybridization was verified using Agilent's 2100 Bioanalyzer (Santa Clara, CA). The Affymetrix (Santa Clara, CA) Mouse Gene 1.0 ST Array was used for the transcript profiling. 20 ng total RNA per sample was processed using WT Ovation Pico RNA Amplification System (NuGEN Technologies, Inc., San Carlos, CA). Sense target cDNAs were generated using the standard NuGEN WT protocol and hybridized to the arrays. Arrays were washed, stained on a FS 450 station and scanned on a GeneChip 3000 7G scanner using GeneChip® Command Console® Software (AGCC).

### Statistical Analysis

Statistical analysis of the CEL files was done using GeneSpring GX 11.5.1 (Agilent Technologies). Signals were quantile-normalized using PLIER16 algorithm and baseline-transformed to the median of all samples. The log2 normalized signal values were filtered to remove entities that show signal in the bottom 20th percentile across all samples. This list was further filtered to only include entities where at least 1 out of 6 conditions have CV<20.0% (that is, to remove probes that are highly variable across replicates in a condition). The list was then subjected to ANOVA (p<0.05) that compares all conditions to the WT-water or WT-DSS saline condition. A Benjamini-Hochberg False Discovery Rate correction (p<0.05) was also included in the analysis. A 1.5-fold filter was applied to identify genes that are differentially expressed between any two specific conditions. Microarray data have been deposited in the Gene Expression Omnibus (GEO) database under accession number GSE32697.

### Real-Time Quantitative PCR Validation of Array Data

Real-time quantitative PCR (RT-qPCR) was performed on colonic total RNA isolated from another set of three mice. To obtain cDNAs, RNAs (2 ug) were reverse-transcribed using the High Capacity RNA-to-cDNA Kit (Applied Biosystems). This was followed by amplification of the undiluted cDNAs with the Fast SYBR Green master mix (Applied Biosystems) and real-time quantification in the StepOne Plus Real-time PCR system. Amplification was performed with 40 cycles of 95°C for 3 sec, and 60°C for 30 sec, using 150 nM of each primer.

The use of *Gapdh* as endogenous control gene in the RT-qPCR validation was problematic with samples that were treated with the recombinant proteins. Preliminary qPCR runs showed fluctuating levels of *Gapdh* among the TSR-treated samples. *Ubc*, *Gapdh*, *Rps23* and *Tpt1* have been evaluated for suitability as internal control in colon cancer studies [16]. Using the GeneMANIA [17] prediction server (www.genemania.org), we found that *Gapdh* interacts with *Igfbp5* (Figure S2). Our array data shows that *Igfbp5* is up-regulated in WT-DSS mice. The *Gapdh*-*Igfbp5* interaction suggests co-expression and could explain the high variability of *Gapdh* expression in our samples. On the other hand, query of *Tpt1* in GeneMANIA showed that it is unrelated to any of our genes of interest.

For the RT-qPCR validation, we selected (1) representative genes from the major pathways that are activated as a response to inflammation, (2) genes that code for acute phase proteins, and (3) genes that have not been extensively characterized, especially in IBD. The genes validated are: calcyphosine-like (*Capsl*), carbonyl reductase 3 (*Cbr3*), CXC chemokine ligand 5 (*Cxcl5*), epiregulin (*Ereg*), haptoglobin (*Hp*), inhibin A (*InhbA*), pentraxin related gene (*Ptx3*), insulin-like growth factor binding protein 5 (*Igfbp5*), matrix metallopeptidase 10 (*Mmp10*, also known as stromelysin-2), *Mmp13*, WAP four-disulfide core domain 12 (*Wfdc12*), serglycin (*Srgn*), Serine (or cysteine) peptidase inhibitor, clade A, member 3N (*Serpina3N*), formyl peptide receptor (*Fpr3*) and leucine-rich repeat-containing G-protein coupled receptor (*Lgr5*). Expression levels of the 15 genes were normalized to the internal control, *Tpt1* (tumor protein, translationally-controlled 1). The 2∧-ddCt method was used to quantify the expression levels of the genes. Details on the reference and target genes are given in the Table S1.

### Immunohistochemistry (IHC)

IHC sections were incubated overnight with the following antibodies: cluster of differentiation 31 (CD31) and Ly-6G/Ly-6C (RB6-8C5) (BD Pharmingen, San Diego, CA), mouse panendothelial cell antigen (MECA-32) (BioLegend, San Diego, CA), Interleukin-6 (IL-6), cluster of differentiation 68 (Mac/CD68) and metalloproteinase 3 (MMP3) (Santa Cruz Biotechnologies Inc. Santa Cruz, CA), LGR5 and Calgranulin MRP8/S100A9 (Epitomics, Burlingame, CA). Sections were further incubated with biotinylated anti-rabbit, anti-goat or anti-rat IgG IMPRESS (Vector Laboratories, Burlingame, CA) respectively for 30 minutes. Finally, color was developed using a 3,3′-diaminobenzidine substrate kit (BD Pharmingen).

### Evaluation of microvascular density (MVD)

MVD was determined in colon sections of the treated mice. Sections stained with CD31/MECA antibodies were screened for colitic lesions. Serial pictures were taken at high power (×400) using a digital camera (Olympus Corporation, Tokyo, Japan), covering both mucosa and submucosa. Images were blindly evaluated; MVD was assessed by counting the vessels in lesions in which endothelial cells were positive for MECA/CD31.

### Evaluation of leukocytic infiltration

Slides stained with antibodies CD68 for macrophages and Ly-6G/Ly-6C for granulocytes were scanned at low magnification and examined for areas with the highest number of leukocytic infiltration. The number of positive cells stained with Mac/CD68 or Ly-6G/Ly-6C was assessed at 400× magnification per field.

## Results

### Only 3TSR ameliorated clinical signs of colitis in the DSS-induced colitis

**Figure 1:**
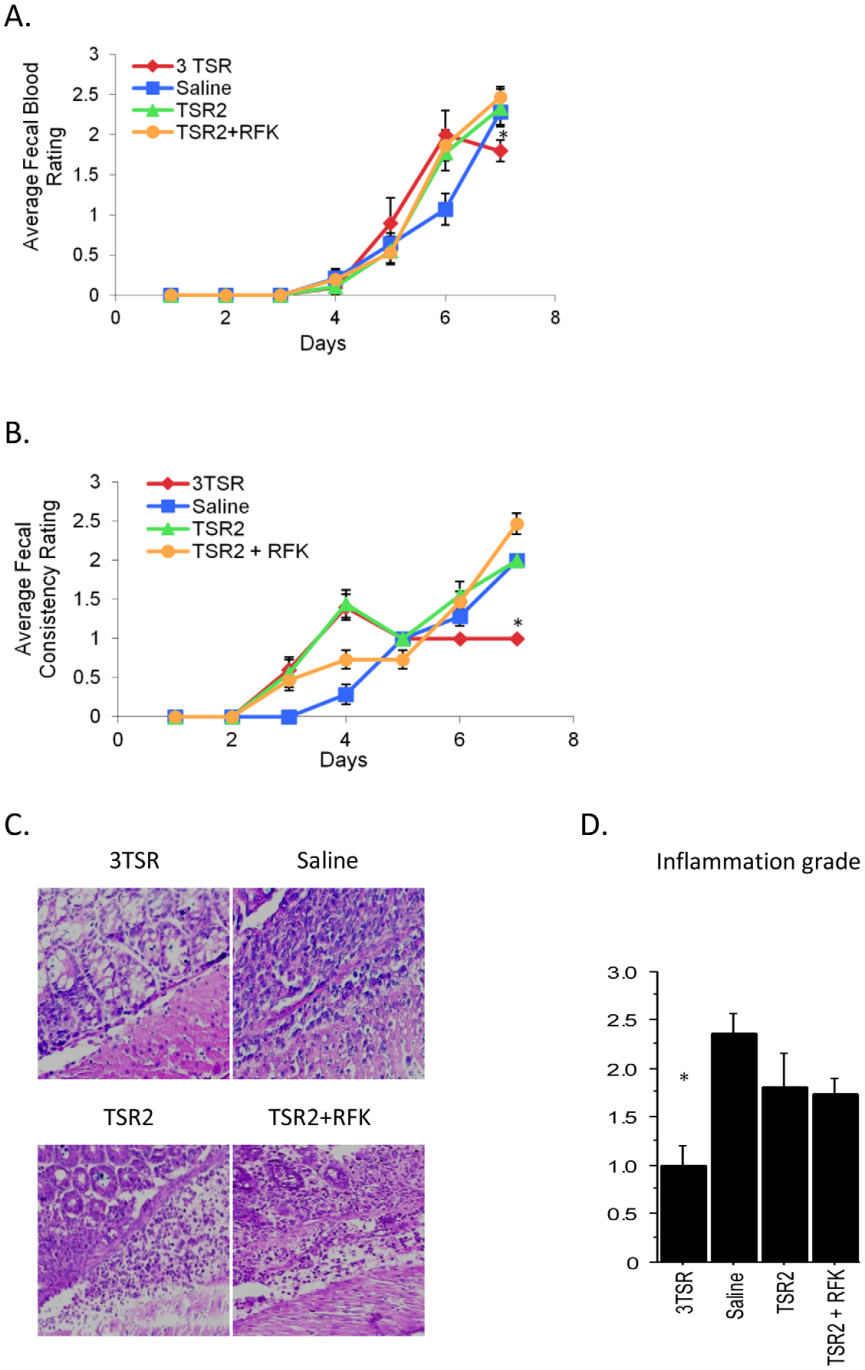
Fecal blood and consistency rating in mice treated with DSS and TSRs over one week. While no significant differences were observed during the first days of treatment, by days 6 and 7, mice treated with the 3TSR (n = 10) showed lesser fecal bleeding when compared with mice treated with the saline (n = 14), TSR2 (n = 9) and TSR2+RFK (n = 15), (day 6: p = 0.02; day 7: p = 0.045, asterisk) (A). Fecal consistency was also evaluated daily (B). By day 7, 3TSR mice displayed more solid feces compared to the liquid feces of saline-treated mice (p<0.0001, asterisk) and TSR2+RFK (p<0.0001). Inflammation grading (C, D) was assessed and H&E stained sections. Decreased inflammation was observed in sections of colons from 3TSR-treated mice (n = 9) compared to colons from mice treated with TSR2+RFK (n = 12), TSR2 (n = 10) and saline (n = 7). 400× original magnification.

The presence of fecal blood was evaluated and rated in mice with DSS-induced colitis and treated with the TSRs (Figure 1A). While no significant differences were observed during the first 5 days of treatment, by days 6 and 7, 3TSR mice showed lesser fecal bleeding when compared with mice treated with saline, TSR2 and TSR2+RFK (day 6, 3TSR and saline p = 0.020; day 7, 3TSR and saline p = 0.045). Fecal consistency was also evaluated daily (Figure 1B). Again, by day 7, 3TSR mice displayed more solid feces compared to the liquid feces of saline-treated mice (p<0.0001) and TSR2+RFK (p<0.0001). TSR2 and TSR2+RFK mice showed more severe diarrhea than the controls (p<0.0001). Inflammation and epithelial injury were significantly decreased in colons of the 3TSR mice when compared with the saline-treated mice (p = 0.001) (Figure 1C). Differences between 3TSR and the other two recombinant proteins were statistically significant (3TSR and TSR2+RFK: p = 0.034; 3TSR and TSR2: p = 0.025) (Figure 1D).

### Angiogenesis was not inhibited by the TSRs

By using CD31/Meca stained slides, endothelial cells lining blood vessels were analyzed in colonic sections (Figure S1) from the treated mice. Vessel counts were not statistically different across the treatments (p = 0.074; 3TSR, n = 29, vessels = 21.48±2.1 and TSR2+RFK, n = 35, vessels = 21.4±1.5). However, colonic sections from TSR2 mice showed a trend for lower counts (n = 20, vessels = 19.45±2.7) when compared to saline-treated colons (n = 16, vessels = 26.62±4.0) (Figure S1).

### Colonic tissues from TSR-treated mice showed differential patterns of leukocytic infiltration

**Figure 2:**
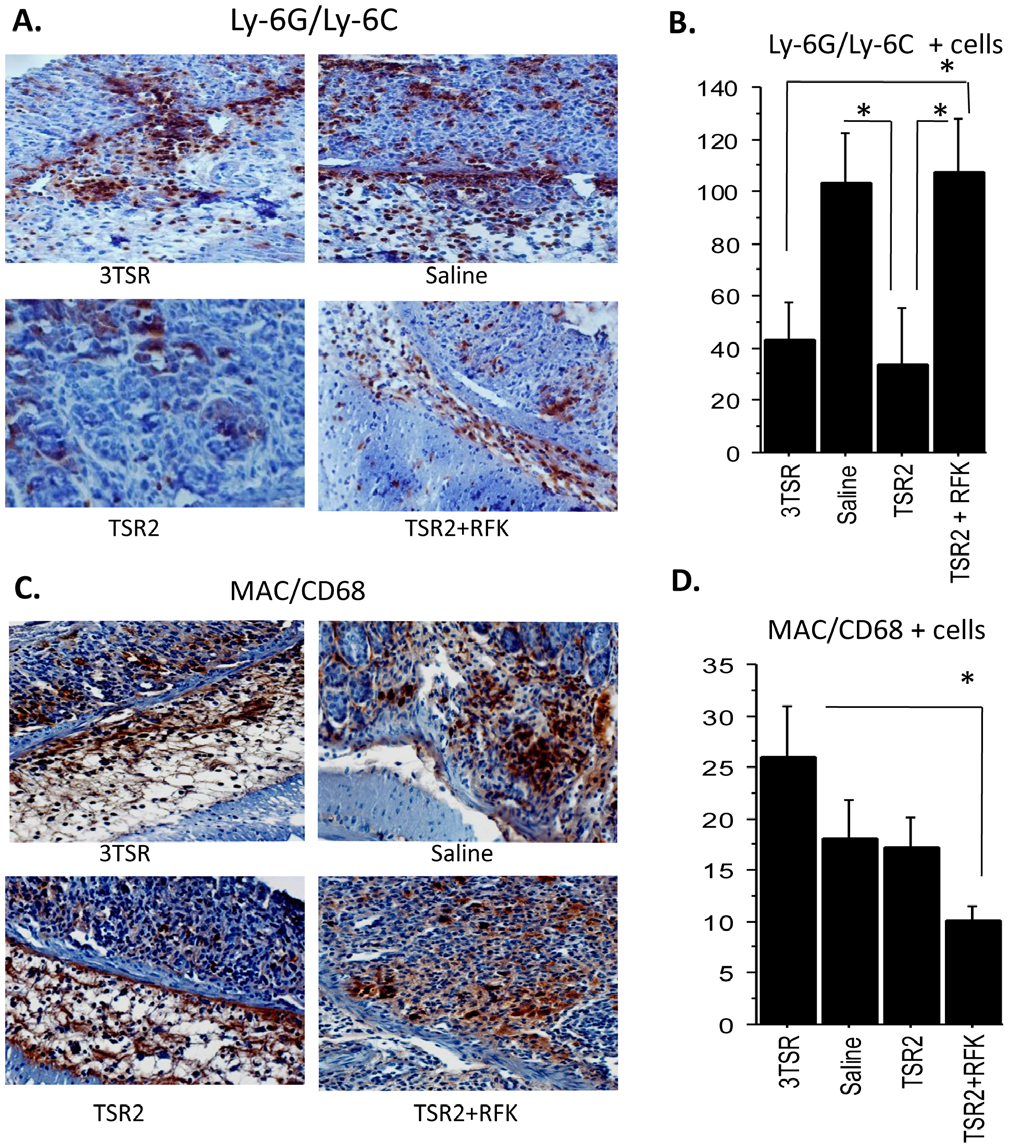
Immunohistochemistry (IHC) with the Ly-6G/Ly-6C antibody for detection of granulocytes and monocytes in colitic sections. Counts of Ly-6G/Ly-6C positive cells in TSR2 and 3TSR were considerably lower than in saline (n = 5 in each group) and TSR2+RFK-treated (n = 9) colons (A). However, only TSR2 showed lower counts that were statistically significant, p = 0.047 (B). IHC against Mac/CD68 (C) showed fewer CD68 positive cells (macrophages) in TSR2+RFK (n = 24) compared to TSR2 (n = 14) and saline (n = 18). These differences were statistically significant, p = 0.0006, when compared with 3TSR-treated (n = 18) colons (D). 400× magnification.

Sections stained with the antibody Ly-6G/Ly-6C were evaluated to identify granulocytes and monocytes (Figure 2A). Colonic sections from 3TSR- and TSR2-treated mice showed counts considerably lower than saline-treated colons, (with p = 0.030 and 0.047, respectively) (Figure 2B). Interestingly, counts obtained from the TSR2+RFK-treated colons were similar to the ones detected in saline-treated colons. The differences between TSR2 and TSR2+RFK were statistically significant (p = 0.021).

Macrophage infiltration was analyzed using Mac/CD68 antibody staining (Figure 2C). Though no differences were detected when each group was compared with the saline control, TSR2+RFK mice showed a significantly lower influx of macrophages compared with the controls and the TSR2 and 3TSR groups (p = 0.0006) (Figure 2D).

### TSR treatment reduced the number of differentially expressed genes in the DSS induced colitis

**Figure 3:**
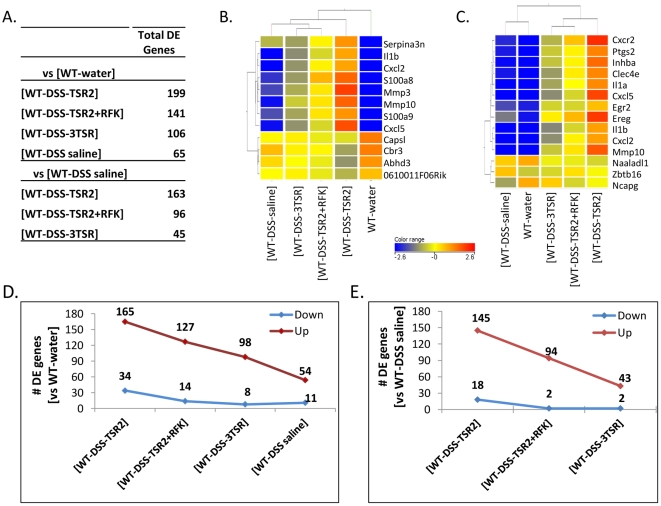
Differentially expressed genes in paired sets of comparison between the treatment and the control groups. Comparison of treatment groups with WT-water (A, upper panel of table) and WT-DSS saline controls (A, lower panel). Total DE genes refer to all genes that are up- or down-regulated by >1.5-fold at 95% confidence level. The 3TSR-treatment group clearly shows the least transcriptional perturbation among the TSR treatment groups. (B) A heatmap of the top 12 DE genes in the comparison that uses WT-water as the control. *Capsl*, *Cbr3*, *Abhd3* and *0610011F06Rik* are down-regulated in the TSR treatment groups. (C) A heatmap of the top DE genes in the 3TSR-treated group. The number of up- and down-regulated genes in the TSR treatment groups (when compared to the WT-water control) are presented in (D); (E) shows the breakdown of the DE genes in comparison to the WT-DSS saline.

Coincident treatment with 3TSR, TSR2+RFK and TSR2 show diminishing numbers of DE genes, with 3TSR showing the least number of DE genes (Figure 3A) when compared to either controls, WT-water or WT-DSS saline. The general trend of number of DE genes (when compared to WT-water) is as follows: WT-DSS-TSR2>WT-DSS-TSR2+RFK>WT-DSS-3TSR>WT-DSS-saline (Figure 3A). A heatmap of the top DE genes, eight up-regulated and four down-regulated, is shown in Figure 3B and the gene list is presented in Table 1. Focusing on the 3TSR, only 43 genes were up-regulated and two genes were down-regulated in the 3TSR vs WT-DSS saline comparison. Figure 3C presents a heatmap of the top 14 DE genes in this pair. The numbers of up- and down-regulated genes in the 3TSR treatment group diminished when compared to either TSR2 or TSR+RFK (Figures 3D and 3E). Table 2 presents the top 12 genes that are up-regulated in the 3TSR-treated group and the two down-regulated genes, using the WT-DSS-saline as reference. Although some genes involved in the inflammatory response were up-regulated in 3TSR, their level of up-regulation was much lower than in the other TSR treatment groups. The complete list of differentially expressed genes (FC>1.5; p<0.1) is provided in the Table S2.

**Table 1:** Top 12 differentially expressed genes (8 up- and 4 down-regulated) in the treatment groups compared to WT-water.

				Fold change (vs [WT-water])				Gene Ontology
Gene Name	Gene symbol	Refseq	Corrected p-value	[WT-DSS saline]	[WT-DSS-3TSR]	[WT-DSS-TSR2]	[WT-DSS-TSR2+RFK]	Biological process//Molecular function//Cellular component
**Up-regulated genes**								
S100 calcium binding protein A9 (calgranulin B)	S100a9	NM_009114	0.026	10.34	18.94	74.93	43.08	leukocyte chemotaxis/actin cytoskeleton reorganization/regulation of integrin biosynthetic process**//**calcium binding
S100 calcium binding protein A8 (calgranulin A)	S100a8	NM_013650	0.031	4.57	9.39	54.98	31.60	chemotaxis**//**calcium ion binding
Chemokine (C-X-C motif) ligand 2	Cxcl2	NM_009140	0.029	4.96	14.01	55.19	31.89	chemotaxis/inflammatory response/immune response/signal transduction**//**cytokine/chemokine activity**//**extracellular region/space
Matrix metallopeptidase 3	Mmp3	NM_010809	0.048	4.95	10.10	69.20	21.49	proteolysis/collagen catabolic process**//**catalytic/metalloendopeptidase/stromelysin 1 activity/calcium/Zn ion binding/hydrolase activity**//**proteinaceous extracellular matrix
Matrix metallopeptidase 10	Mmp10	NM_019471	0.036	4.54	17.11	74.14	29.54	proteolysis/metabolic/collagen catabolic process**//**catalytic/metalloendopeptidase activity/calcium/Zn binding/hydrolase/stromelysin 2 activity**//**proteinaceous extracellular matrix
Interleukin 1 beta	Il1b	NM_008361	0.030	3.07	9.59	39.65	15.96	fever/inflammatory/immune response/cell proliferation/neutrophil chemotaxis/positive regulation of I-kappaB kinase/NF-kappaB cascade/positive regulation of chemokine (IL-6) biosynthetic process/positive regulation of JNK cascade/leukocyte migration**//**IL-1 receptor binding/cytokine, growth factor activity**//**extracellular region/space
Chemokine (C-X-C motif) ligand 5	Cxcl5	NM_009141	0.030	1.30	3.33	24.56	5.37	chemotaxis/inflammatory/immune response/signal transduction**//**cytokine/chemokine activity**//**extracellular region/space
Serine (or cysteine) peptidase inhibitor, clade A, member 3N	Serpina3n	NM_009252	0.030	4.92	4.22	17.01	8.73	acute-phase response**//**serine-type endopeptidase inhibitor activity**//**extracellular region/space
**Down-regulated genes**								
Carbonyl reductase 3	Cbr3	NM_173047	0.044	−1.54	−2.22	−4.64	−2.31	metabolic process**//**carbonyl reductase (NADPH) activity/oxidoreductase activity**//**cytosol
Abhydrolase domain containing 3	Abhd3	NM_134130	0.046	−1.62	−2.13	−4.42	−2.43	no biological data available**//**carboxylesterase activity/hydrolase activity**//**integral to membrane
RIKEN cDNA 0610011F06 gene	0610011F06Rik	NM_026686	0.047	−1.55	−1.44	−2.63	−1.81	---**//**methyltransferase activity/transferase activity
Calcyphosine-like	Capsl	NM_029341	0.028	−2.41	−2.26	−3.20	−2.93	---**//**calcium ion binding**//**cytoplasm

**Table 2:** Top 14 differentially-expressed genes in the 3TSR group.

				Fold change (vs [WT-DSS saline])				
Gene name	Gene symbol	Refseq	Corrected p-value	[WT-DSS-3TSR]	[WT-DSS-TSR2+RFK]	[WT-DSS-TSR2]	[WT-water]	Pathway or {Gene Ontology}*
**Up-regulated**								
Interleukin 1 alpha	Il1a	NM_010554	0.036	3.84	6.97	15.18	−1.41	Inflammatory_Response_Pathway
Matrix metallopeptidase 10	Mmp10	NM_019471	0.036	3.77	6.51	16.33	−4.54	Matrix_Metalloproteinases
Inhibin beta-A	Inhba	NM_008380	0.038	3.45	5.57	13.86	−1.40	TGF_Beta_Signaling_Pathway
Prostaglandin-endoperoxide synthase 2	Ptgs2	NM_011198	0.034	3.16	4.90	10.37	−1.59	Eicosanoid_Synthesis///Prostaglandin_synthesis_regulation
Interleukin 1 beta	Il1b	NM_008361	0.030	3.13	5.21	12.94	−3.07	Inflammatory_Response_Pathway///Smooth_muscle_contraction
Chemokine (C-X-C motif) ligand 2	Cxcl2	NM_009140	0.029	2.82	6.43	11.12	−4.96	{inflammatory response/immune response//leukocyte/neutrophil chemotaxis///extracellular}
Epiregulin	Ereg	NM_007950	0.039	2.68	3.84	8.80	−1.98	{angiogenesis///positive regulation of cytokine production//epidermal growth factor receptor signaling pathway}
Chemokine (C-X-C motif) ligand 5	Cxcl5	NM_009141	0.030	2.56	4.14	18.92	−1.30	{cytokine production///chemotaxis///inflammatory response///immune response}
Non-SMC condensin I complex, subunit G	Ncapg	NM_019438	0.045	2.56	1.40	1.75	3.21	{no biological data available}
Chemokine (C-X-C motif) receptor 2	Cxcr2	NM_009909	0.040	2.55	6.18	16.62	−1.94	{apoptosis///chemotaxis///signal transduction///G-protein coupled receptor protein signaling pathway}
Early growth response 2	Egr2	NM_010118	0.038	2.45	2.49	5.05	−1.34	{regulation of ossification///response to insulin stimulus///positive regulation of transcription from RNA polymerase II promoter}
C-type lectin domain family 4, member e	Clec4e	NM_019948	0.044	2.42	5.24	10.30	−1.51	{integral to membrane///sugar binding}
**Down-regulated**								
N-acetylated alpha-linked acidic dipeptidase-like 1	Naaladl1	NM_001009546	0.045	−1.55	−1.59	−1.84	1.26	{proteolysis///metallopeptidase activity///integral to membrane}
Zinc finger and BTB domain containing 16	Zbtb16	NM_001033324	0.038	−1.95	−1.22	−1.55	−2.13	{hemopoiesis///transcriptional repressor complex///protein C-terminus binding}

* The gene ontology is given, enclosed in brackets, where pathway has not been annotated in the microarray datasheet.

### DSS and the TSR treatments up-regulated key genes involved in inflammation, cell adhesion and apoptosis

Genes up-regulated in the DSS and TSR-treatment groups belong to the following pathways: (1) matrix metalloproteinases, (2) inflammatory response, (3) peptide GPCRs, (4) eicosanoid/prostaglandin synthesis regulation, (5) TGF-beta signaling, (6) smooth muscle contraction, (7) integrin-mediated cell adhesion and (8) apoptosis (Table 3). S100 calcium binding protein A9 (*S100a9*; also known as calgranulin B) had the highest transcript level in all treatment groups, with the WT-DSS group showing the highest FC (81), whereas the WT-DSS-3TSR has FC = 18 (Table S2). An inflammatory response was indicated by the high transcript levels of *Il-6* [WT-DSS-TSR2 (FC = 18)>WT-DSS-TSR2+RFK (FC = 7)>WT-DSS-3TSR (FC = 4)]. Chemokines, specifically *Cxcl2*, *Cxcl5*, *Cxcl13*, *Cxcl16*, *Ccl3* and *Ccl7*, were also up-regulated (FC = 2 to 60) in the treated samples. *Cxcl2*, in particular, had the greatest increase in the WT-DSS-TSR2 (FC = 55) compared to the other TSR-treated mice. Genes involved in inflammation and angiogenesis including *Adamts9*, *Tnf* alpha and the TNF receptor *Tnfrsf1b*, were enriched the most in the WT-DSS and WT-DSS-TSR2 groups.

**Table 3:** Major pathways activated and genes enriched in the DSS- and TSR-treated mice.

Pathways	Genes up-regulated (fold change>2.0, p<0.05)
**Matrix metalloproteinases**	Matrix metallopeptidases 2, 3, 8, 10, 12, 13, 14
**Inflammatory response**	Interleukin 1 alpha (*IL1a*), *IL1â*, *IL-6*, Interleukin 4 receptor, alpha (*Il4ra*), *Il1r2*
**Peptide GPCRs**	Formyl peptide receptor 3 (*Fpr3|2*)
**Eicosanoid/prostaglandin synthesis regulation**	Prostaglandin-endoperoxide synthase 2 (*Ptgs2*)
**TGF-beta signaling**	Inhibin beta-A (*Inhba*)
**Smooth muscle contraction**	Insulin-like growth factor binding protein 5 (*Igfbp5*)
**Blood clotting cascade/Wnt signaling**	Plasminogen activator, urokinase (*Plau*)
**Integrin-mediated cell adhesion**	Integrins alpha M (*Itgam*), beta 2 (*Itgb2*), *Itga4*
**Apoptosis**	B-cell leukemia/lymphoma 2 related protein A1a (*Bcl2a1a*), *Bcl2a1b*, *Bcl2a1c*, *Bcl2a1d*; Tumor necrosis factor (*Tnf*), TNF receptor superfamily, member 1a (*Tnfrsf1a*), *Tnfrsf1b*
**Ovarian Infertility Genes**	Early growth response 2 (*Egr2*),CCAAT/enhancer binding protein (*C/EBP*), beta (*Cebpb*)

Genes coding for proteins that are well known to interact with TSP-1 during inflammation were differentially expressed in DSS and TSR-treated mice, except in the 3TSR group. *Cd47* was only slightly up-regulated in WT-DSS-TSR2 (1.8). It was not DE in TSR2+RFK and 3TSR. *Tgfβ1* was also slightly up-regulated in TSR2 (FC = 2.5) and TSR2+RFK (FC = 1.8), but not in 3TSR. None of the treatment samples showed differential expression of *Cd36*.

### Real-time qPCR validation

**Figure 4:**
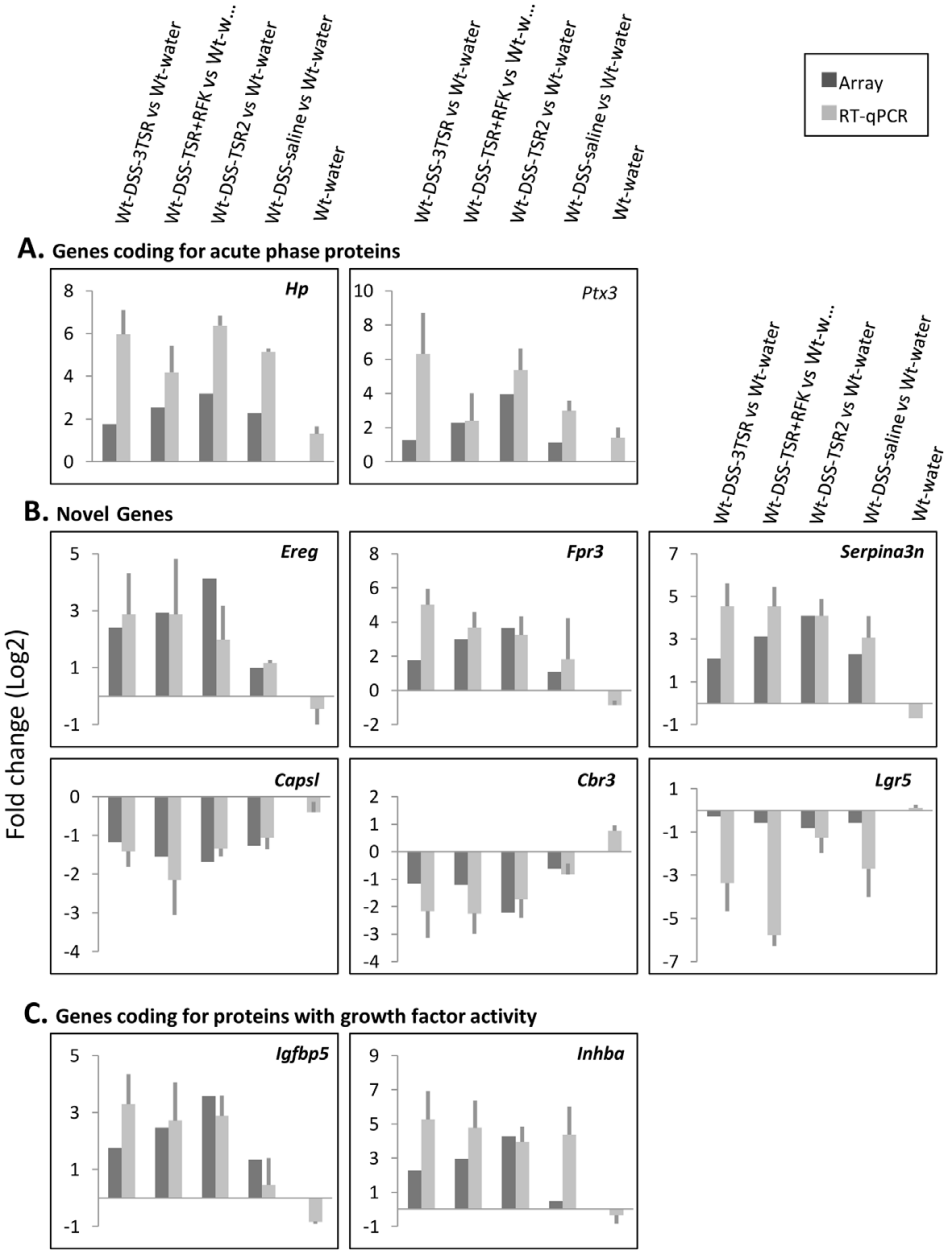
SyBR Green-based RT-qPCR validation of array data. Signal intensities were normalized to the endogenous control, *Tpt1*. Fold changes are the averages of three biological replicates different from those used in the microarrays. Linear FC values were log2-transformed. *Hp* and *Ptx3* code for acute phase proteins (A). We classified *Capsl*, *Cbr3*, *Ereg*, *Fpr3*, *Serpina3n* and *Lgr5* as novel genes (B) because they have not been characterized in IBD. Growth factors are clearly up-regulated (C).

Actual qPCR data revealed no fluctuation in expression of the internal control gene, *Tpt1*, which was consistent with the microarray data. We validated 15 DE genes to verify the array data. Figure 4 summarizes the validation for representative genes coding for acute phase proteins, growth factors and novel proteins. Validation for the metalloproteinases and genes coding for cytokines and a protein important for intestinal homeostasis are shown in Figure S3. Although *Srgn* and *Wfdc12* have p-values greater than 0.05, (*Srgn* p = 0.056, *Wfdc12* p = 0.065), the real-time qPCR validation shows that they are indeed differentially expressed.

### IHC validation of genes recognized in stem cell biology, cancer and inflammation

Array data show that *Lgr5*, an intestinal stem cell biomarker [18], [19], was down-regulated in the TSR2-treated WT mice. *Lgr5* was not differentially expressed in the 3TSR and TSR2+RFK mice. However, results obtained in the RT-qPCR validation show that *Lgr5* was significantly down-regulated in the TSR2+RFK (Figure 4B).

**Figure 5:**
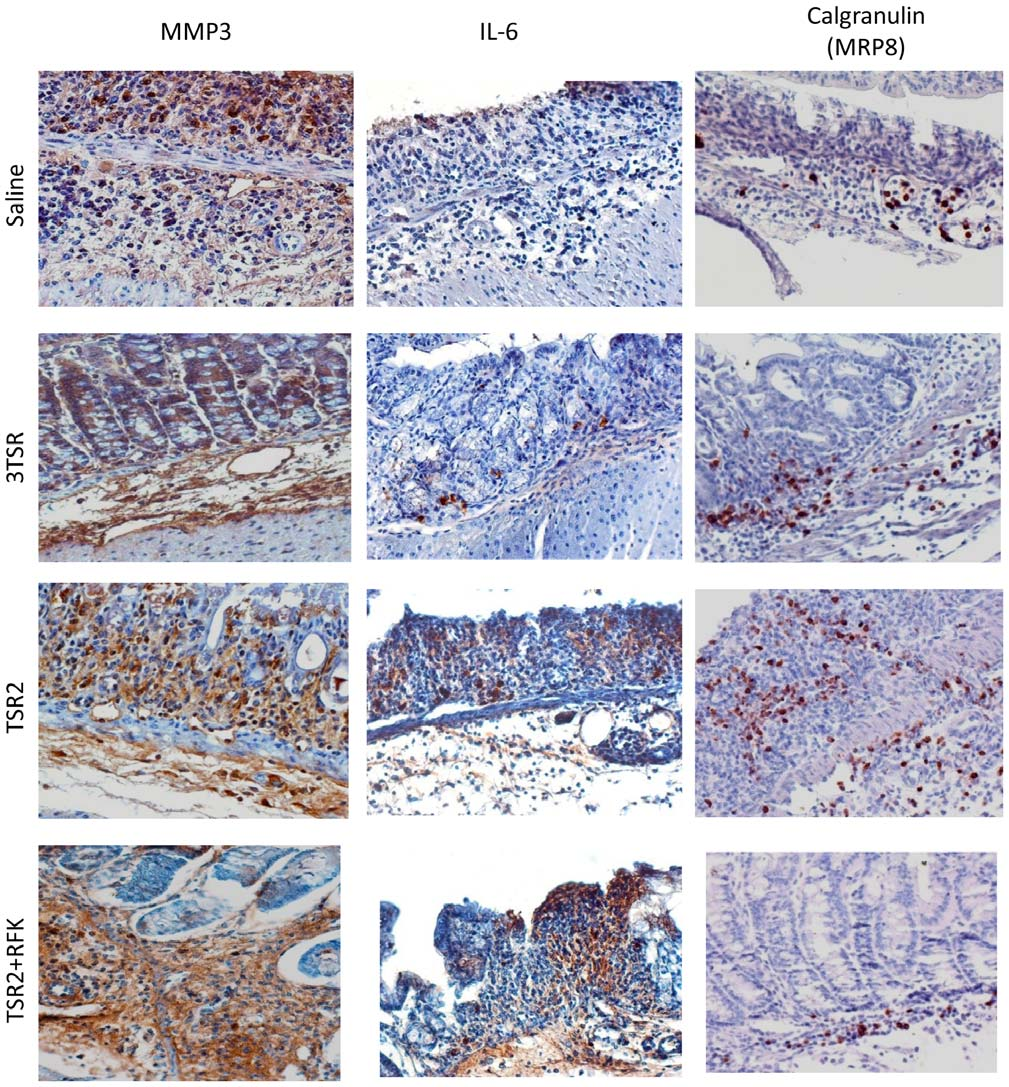
IHC of MMP3, IL-6 and MRP8. Protein expression of MMP3 was localized in colonocytes, leukocytes and endothelial cells. Staining was particularly intense in the submucosal stroma of colitic areas. IL-6 was detected in the leukocytic infiltrate and endothelial cells. Calgranulin or MRP8 also showed selective staining in the leukocytic infiltrate.

MMP3, IL-6 and Calgranulin (MRP8) are relevant in angiogenesis and inflammation. Validation by IHC of these genes (Figure 5) showed the MMP3 protein localized in the cytoplasm of epithelial cells and endothelial cells. Strong positive staining for MMP3 was detected mainly in monocytes, polymorphonuclears, plasma and fibroblasts. Stromal staining was detected in the submucosa. Endothelial cells and the leukocytic infiltrate in inflamed areas were positive for IL-6. MRP8 staining was observed almost exclusively in polymorphonuclears.

## Discussion

Our results indicate that 3TSR suppressed the inflammatory response induced by DSS injury and these data are supported by the array results. For pharmacological consideration, it is logical to assume that the lesser the perturbation of the transcriptome, the better the clinical outcome. Our results also confirm the finding that TSR2 is less effective than either 3TSR or TSR2+RFK [10]. 3TSR has been shown to induce endothelial cell apoptosis and inhibit tumor growth [11]. This function may be correlated to the modulation of VEGFR2 function by TSP-1 and 3TSR [20].

One of the unexpected findings when clinical parameters were evaluated was the severity of the bleeding and diarrhea in mice treated with TSR2 and TSR2+RFK. These results do not agree with previous studies using the ABT-510, a TSR2 mimetic peptide [15]. In the ABT-510 studies, osmotic mini-pumps were used for the continuous delivery of the peptide in contrast to the daily injections used in this study. Moreover, increased eNOS activity has been attributed to increased mucosal blood flow in induced colitis [21] and TSP-1 is known to regulate nitric oxide (NO) signaling [22]. Noteworthy is the lesser influx of granulocytes in the TSR2 mice compared to the TSR2+RFK and the 3TSR. This suggests that the TSR2 domain inhibits the influx of granulocytes independently of the RFK domain. TSR2 has been demonstrated to inhibit the secretion of NO during the acute phase of inflammation, and therefore decreasing the trafficking of neutrophils [23]. On the other hand, RFK presumably inhibits inflammation by activating TGFß1. However, recent studies suggest that when there is a high secretion of IL-1 and IL-6, TGFβ1 will favor the production of Th17 cells. Th17 cells secrete pro-inflammatory cytokines and metalloproteinases that exacerbate the inflammatory response [23].

Our array data showed that metalloproteases and chemokines such as MMP10, MMP13, the CCs and CXCs are involved in the pathogenesis of DSS colitis. Expression of *Cxcl2* and *Cxcl5* (Figure S3B), the proteins of which are known to target neutrophils and have suspected roles in IBD, was significantly increased in the DSS and TSR-treated mice during the acute phase of colitis. The increase in *Cxcl2* expression is the same observation reported for the leptin receptor-deficient mice that were treated with DSS [24]. An irregularity in chemokine signaling could jeopardize epithelial integrity, which could lead to the breakdown of defense against invading pathogens. Moreover, *Cxcl5* has a great potential in eliciting a pro-angiogenic phenotype [25].

The concentration of acute phase proteins is known to increase or decrease during inflammation. *Hp* and *Ptx3*, both of which are enriched in the DSS and TSR-treated mice, code for acute phase proteins. In a study of Hungarian patients with Crohn's disease (CD), *Hp* polymorphism was determined to be associated with CD and inflammatory disease behavior [26]. Hp has been suggested to have a protective role in inflammatory colitis [27]. *Ptx3* has been annotated to “positive regulation of nitric oxide biosynthetic process” and to “zymosan binding”.

This study showed strong evidence for the alteration of gene expression in DSS-induced colitis that supports vulnerability to cancer. As an example, calgranulins, S100A8/A9 (calgranulins A and B, respectively), have been detected in various human cancers; hence they have been suggested to play a key role in inflammation-associated cancer [28]. They support epithelial barrier function and mediate responses to infection and inflammation [29] by amplifying pro-inflammatory responses [30]. In gastrointestinal epithelial cells, their expression is induced during inflammation and increased protein expression of S100A9 has been detected in colitis-related carcinogenesis in mice [31]. Key genes associated with colon tumor progression are reportedly activated by S100A8/A9 [32].

The ‘novel’ genes that we validated appear to have a role, one way or another, in inflammatory response and the promotion of inflammation-related cancer development. The function of these genes as they relate to diseases and how these genes interact with other genes have not been extensively studied. The description ascribed to them in the gene ontology database, however, indicates some involvement in the development of physiological disorders. Genome-wide association studies have identified *Capsl* to have association with type 1 diabetes [33] and the autoimmune thyroid disease Graves' disease [34]. *Cbr3*, an enzyme that reduces carbonyl groups in xenobiotics and endogenous compounds such as steroids and eicosanoids, has been suggested to have a minor role in xenobiotic metabolism in humans [35]. *Ereg* is a growth factor that resides in the extracellular space and is a marker for ovarian cancer development [36]. *Fpr3* is a member of a G-protein coupled receptor family that controls immune responses such as leukocyte chemotaxis and activation [37]. *Serpina3n* has been found to inhibit human Granzyme B activity in sertoli cells as a mechanism to suppress the immune response [38].

SRGN is a proteoglycan that has important roles in inflammatory reactions [39]. In human endothelial cells, it co-localizes and interacts with tissue-type plasminogen activator (PLAT) [40]. In macrophages, where it is the major secreted proteoglycan, it regulates the secretion of TNF [41]. The up-regulation of *Srgn* (Figure S3B) in the DSS- and TSR-treated mice suggests that it is a component of the immune response in these mice.

The significance of *Wfdc12* up-regulation in the DSS- and TSR-treated mice (Figure S3C), lies in the fact that an altered microbiota composition in the intestinal tract characterizes the pathogenesis of IBD [42], [43]. WFDC12 is an antibacterial protein that inhibits the growth of both *E. coli* and *S. aureus* at a IC90 of 10 µM [44]. With its anti-infective activity, it could serve as mucosal defense against pathogenic bacterial strains, in conjunction with chemokine signaling.

Evidence for involvement of Insulin-like growth factor (IGF) signaling in cancer progression has been increasing [45]. IGF signaling plays a critical role in promoting cell survival and proliferation of embryonic and adult stem cells [46]. IGFBP5 is associated with “stemness” and its expression has been linked to LGR5, which has been shown to be expressed in cycling columnar epithelial cells at the intestinal crypt base [18], [19]. LGR5-deficient mice showed decreased *Igfbp5* expression [47]. Erosive lesions in the intestinal epithelium due to DSS could account for the lower transcript levels of *Lgr5* in the DSS-treated mice. LGR5 deficiency was reported to deregulate the Wnt pathway and contribute to cancer [47].

In conclusion, this study showed that the 3TSR have anti-inflammatory properties that seem to be independent of any angiogenic inhibition or ability to activate TGFß1. These data highlight the anti-inflammatory functions of the 3TSR of TSP-1 in the mouse intestine and its potential use as novel therapeutic tool in IBD. The transcript profiles of the treatment groups showed a pool of genes involved in critical pathways of inflammation. The identification of these genes has implications in improving our understanding of the pathophysiology of IBD. Further characterization of their protein products and their interactions could provide leads in the development of other drug therapy for IBD.

## Supporting Information

Figure S1 **Assessment of microvessel counts.** Using combined MECA/CD31 antibodies (A), fewer blood vessels were observed in sections from TSR2 mice (n = 20) compared to sections from mice treated with TSR2+RFK (n = 35), 3TSR (n = 29) or saline (n = 16), (B). 400× magnification.
(TIF)

Figure S2 **A functional association network of selected mouse genes.** GeneMANIA, a web interface for predicting gene function and determining usability of genes for functional assays (www.genemania.org) was used to determine suitability of endogenous control genes for validating array data by RT-qPCR. The following genes (grey circles) were inputted in GeneMANIA: *Ubc*; *Tpt1*; *Rps23*; *Gapdh*; *Capsl*; *Igfbp5*; *Fpr3*; *Srgn*; *Kcne3*; *Mmp10*; *Mmp13*; *Wfdc12*. The network generated shows interaction between *Gapdh* and *Igfbp5*; therefore, *Gapdh* expression is likely to be co-expressed with *Igfbp5* and show varying expression levels similar to that of *Igfbp5*. *Tpt1*, on the other hand, has no predicted interaction with any of the input genes.
(TIF)

Figure S3 **Additional RT-qPCR validation of array data.**
(TIF)

Table S1 **Genes used for the RT-qPCR validation and their primers.** *Tpt1* was used as the control gene after it was found to show no fluctuation compared to four other genes that were tested.
(XLSX)

Table S2 **ANOVA list of genes that were differentially expressed in the colons of DSS and TSR-treated mice.**
(XLS)
