# Supplementary figures and images for: Thrombospondin-1 Type 1 Repeats in a Model of Inflammatory Bowel Disease: Transcript Profile and Therapeutic Effects
Source: PLoS One. 2012 Apr 3;7(4):e34590. doi: 10.1371/journal.pone.0034590 (PMC3318003; doi:10.1371/journal.pone.0034590)

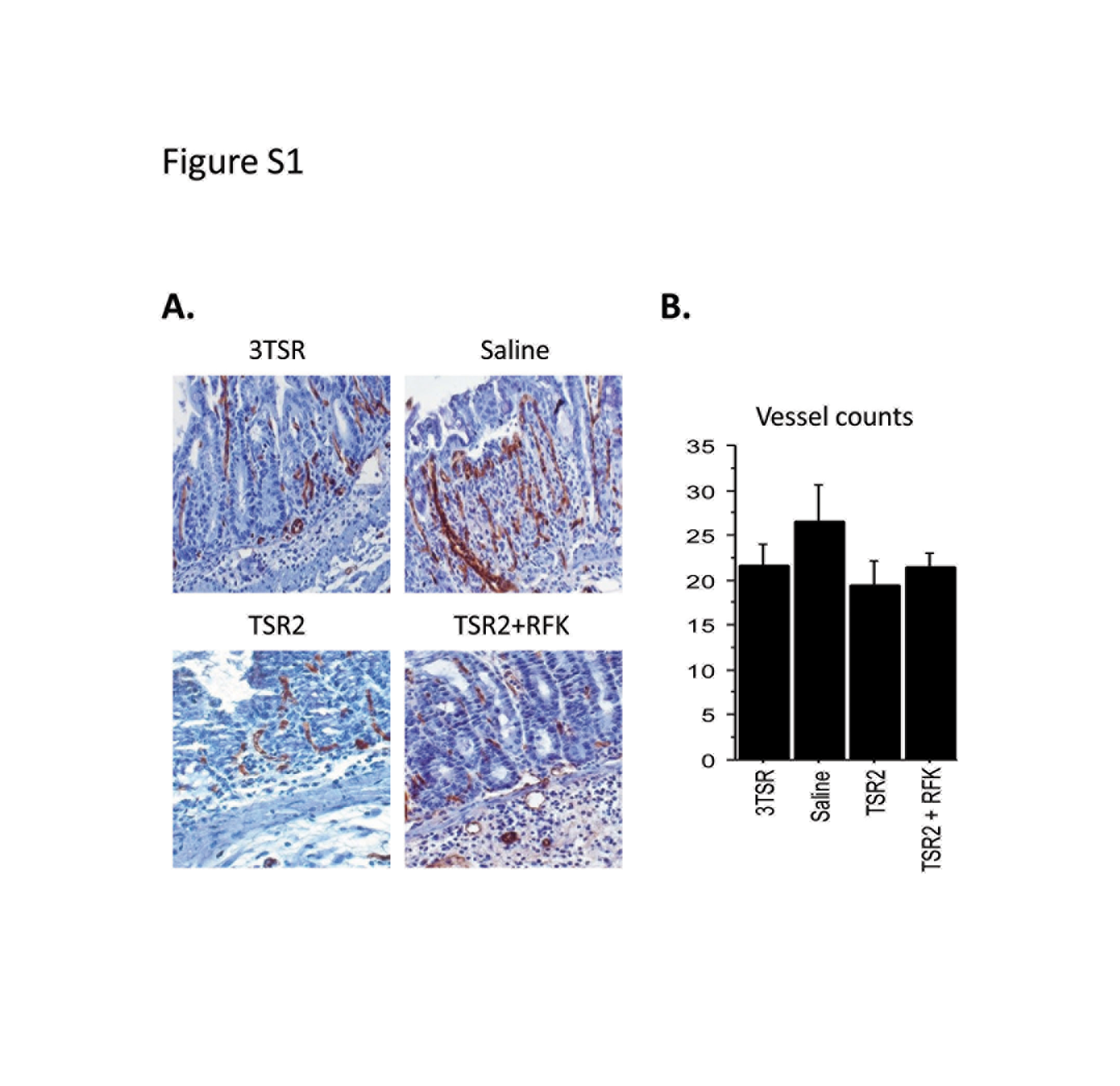

Supplement: Figure S1 — Assessment of microvessel counts. Using combined MECA/CD31 antibodies (A), fewer blood vessels were observed in sections from TSR2 mice (n = 20) compared to sections from mice treated with TSR2+RFK (n = 35), 3TSR (n = 29) or saline (n = 16), (B). 400× magnification. (TIF) [file pone.0034590.s001.tif]

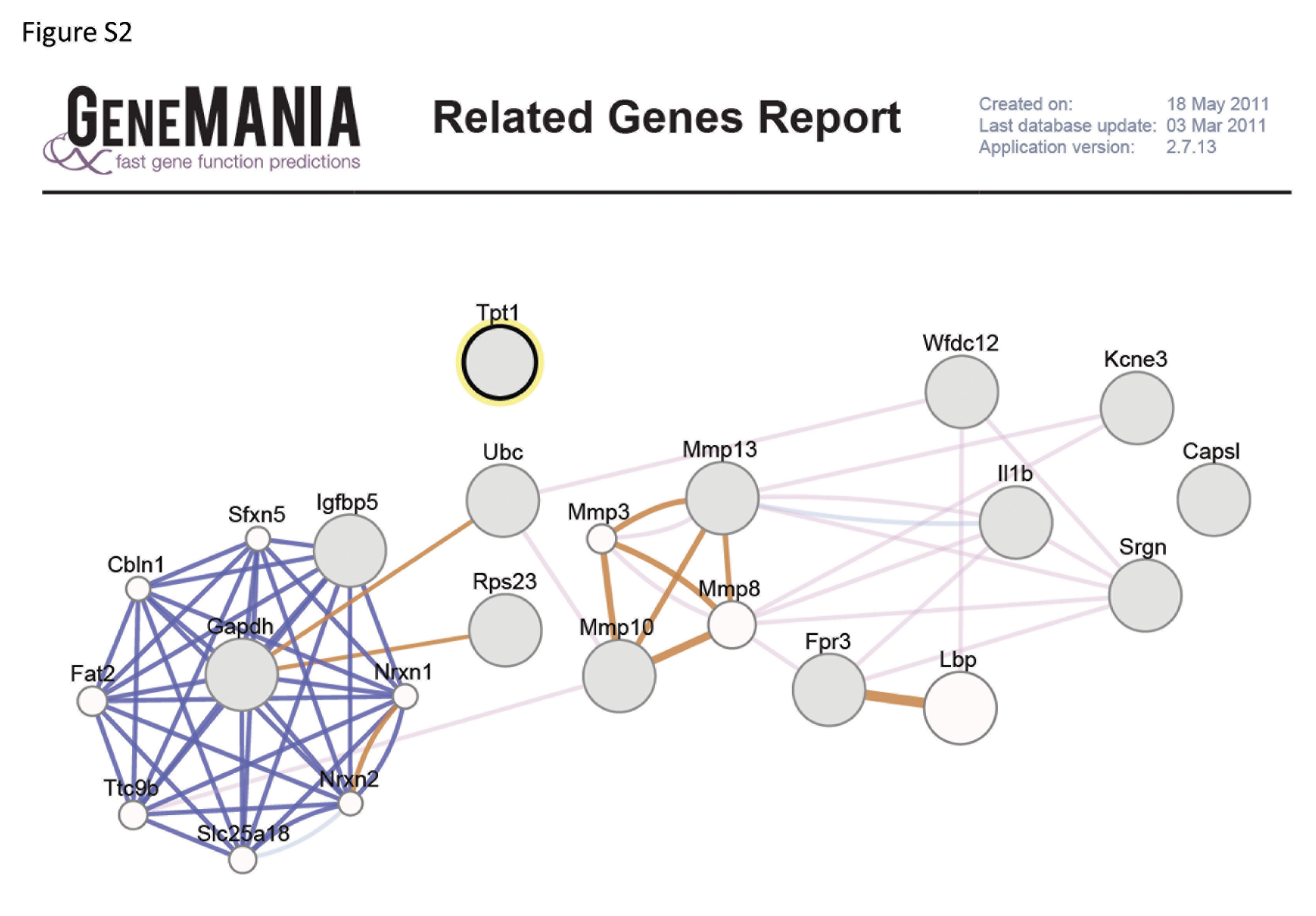

Supplement: Figure S2 — A functional association network of selected mouse genes. GeneMANIA, a web interface for predicting gene function and determining usability of genes for functional assays (www.genemania.org) was used to determine suitability of endogenous control genes for validating array data by RT-qPCR. The following genes (grey circles) were inputted in GeneMANIA: Ubc; Tpt1; Rps23; Gapdh; Capsl; Igfbp5; Fpr3; Srgn; Kcne3; Mmp10; Mmp13; Wfdc12. The network generated shows interaction between Gapdh and Igfbp5; therefore, Gapdh expression is likely to be co-expressed with Igfbp5 and show varying expression levels similar to that of Igfbp5. Tpt1, on the other hand, has no predicted interaction with any of the input genes. (TIF) [file pone.0034590.s002.tif]

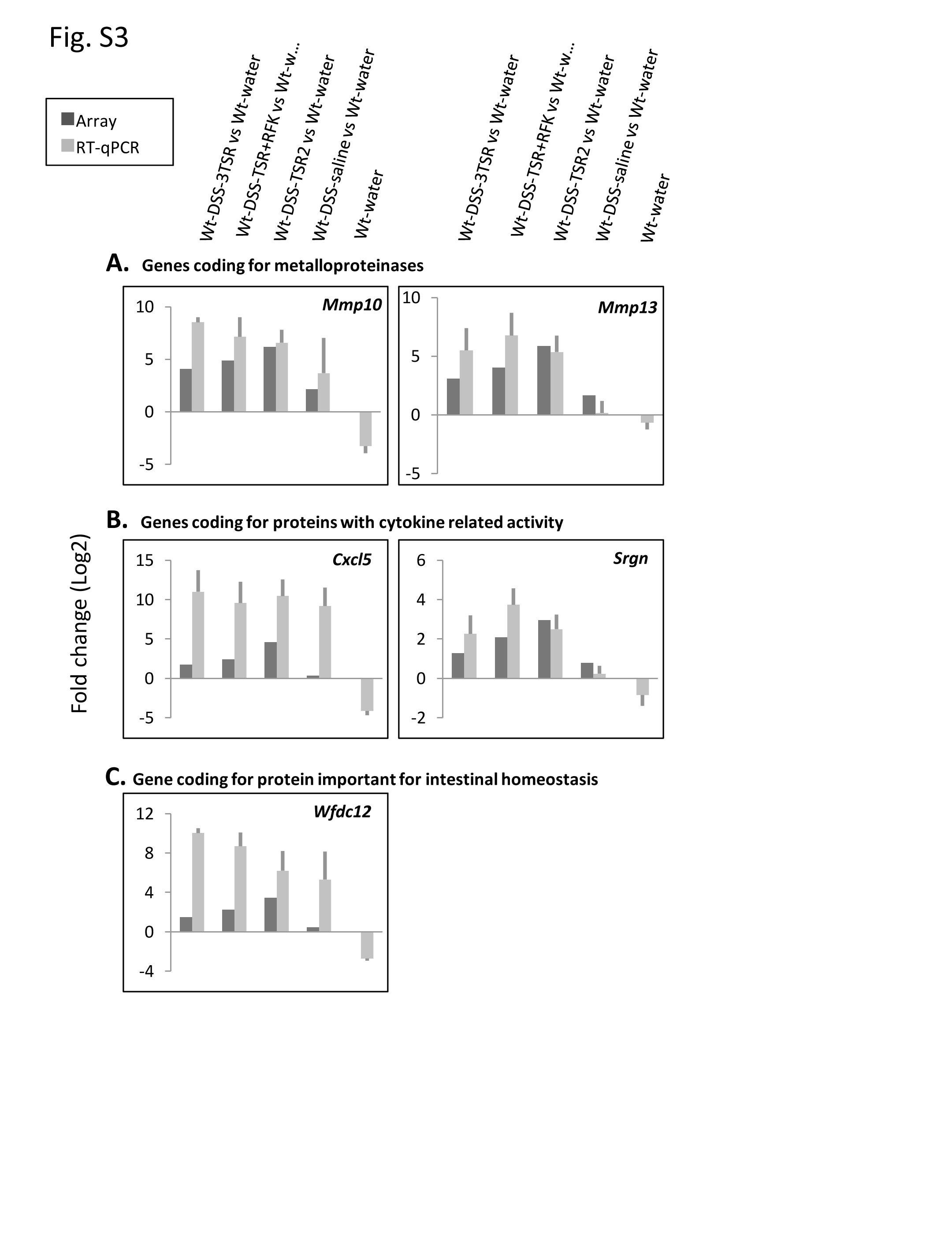

Supplement: Figure S3 — Additional RT-qPCR validation of array data. (TIF) [file pone.0034590.s003.tif]
